# Supplementary material for: An Intrabody against B-Cell Receptor-Associated Protein 31 (BAP31) Suppresses the Glycosylation of the Epithelial Cell-Adhesion Molecule (EpCAM) via Affecting the Formation of the Sec61-Translocon-Associated Protein (TRAP) Complex
Source: Int J Mol Sci. 2023 Sep 30;24(19):14787. doi: 10.3390/ijms241914787 (PMC10572819; doi:10.3390/ijms241914787)
Supplement: Supplementary file 1 [file ijms-24-14787-s001.zip › ijms-2560687-supplementary.pdf]

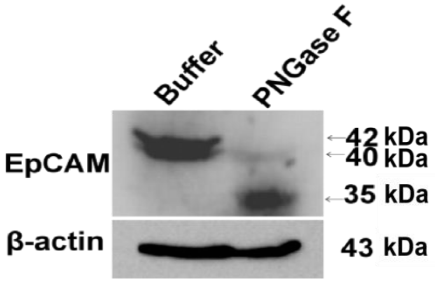

Figure S1. Glycosidase treatment. Treatment of EpCAM with Peptide-N-Glycosidase F (PNGase F) at 37℃ for 4 h. Cell lysates derived from MKN-45 cells were resuspended in 0.5% SDS and 40 mM DTT. Following boiling for 10 min, samples were added to GlycoBuffer with 1% NP40 and digested with PNGase F as indicated.

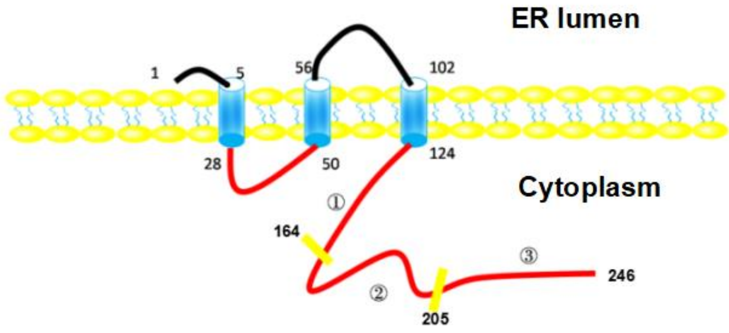

Figure S2. Diagrammatic sketch of BAP31. The N-terminal half of these proteins is membrane-bound, while the C-terminal half is cytoplasmic. The C-terminus of BAP31 in the cytoplasm was divided into three sections (124-164 aa), (164-205 aa) and (206-246 aa). The antibody against (164-205 aa) was screened from the human phage single domain antibody library.

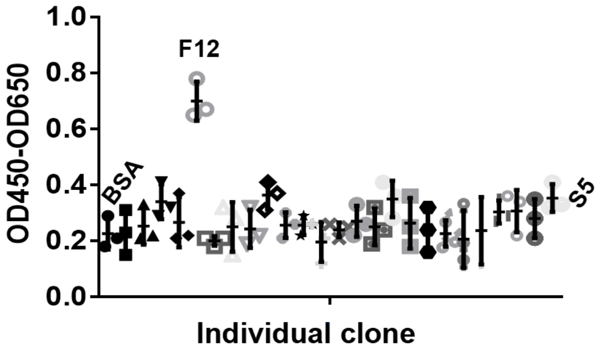

Figure S3. Binding of VH fragments to BAP31. Individual clone picked from the fourth round of panning was induced in TG-1 bacteria by IPTG to produce soluble VH fragments. Binding of VH fragments to BAP31 was analyzed by ELISA. Signals from BSA-coated plates were used as a negative control. Signals from VH-irrelevant (S5) were used as an antibody control.

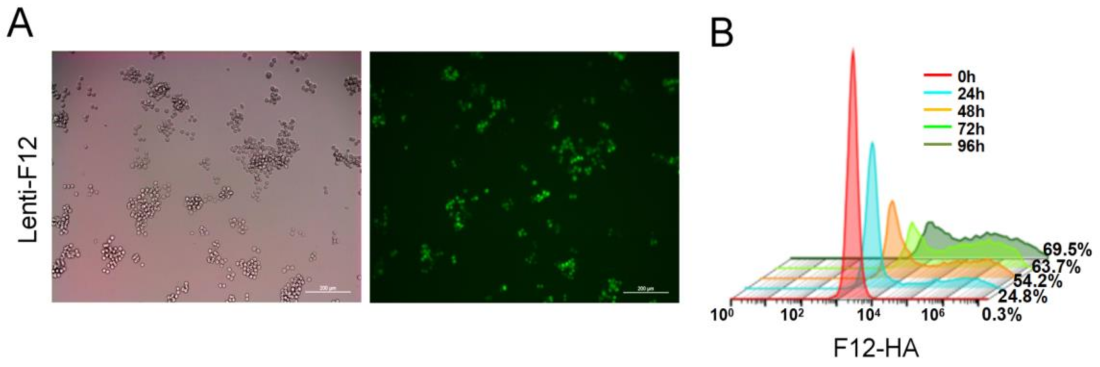

Figure S4. Expression efficiency of VH-F12 intrabody in GC cells. (**A**) MKN-45 cells were transduced with 50 MOI of Lenti-VH-F12. Green fluoresce intensity observed by fluorescence microscope, and the transduction efficiency was greater than 70% in MKN-45 cells following 72 h of transduction (scale bar 200 μm). (**B**) MKN-45 cells were transfected with pcDNA3.1(-)-VH-F12. Following 0, 24, 48, 72 and 96 h of transfection, the transfection efficiency of pcDNA3.1(-)-VH-F12 was examined through flow cytometry analysis using a monoclonal antibody against HA, followed by FITC-conjugated secondary antibodies.

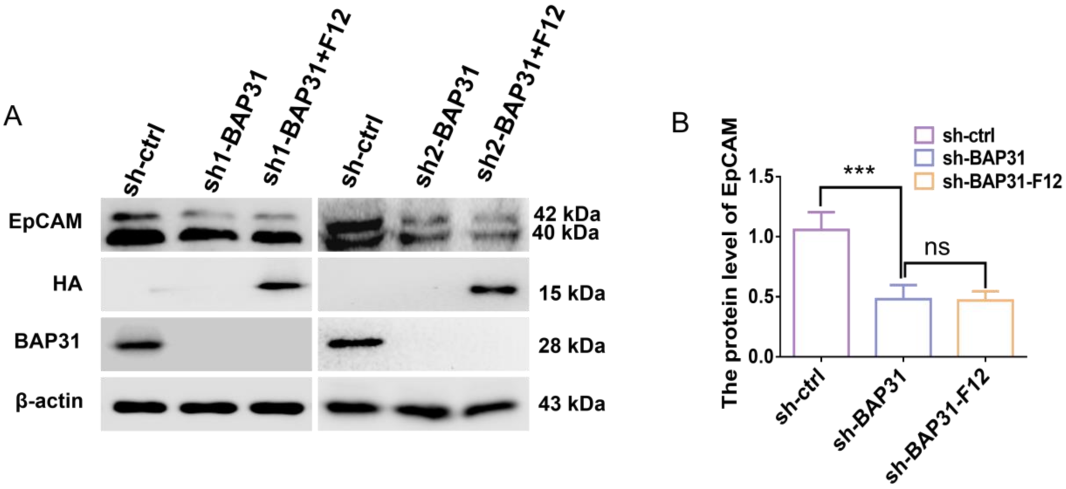

Figure S5. The effect of VH-F12 on EpCAM in BAP31 stable knockout MKN-45 cells. (**A**) The effect of VH-F12 on N-glycosylation of EpCAM in BAP31 knockout MKN-45 cells was investigated. BAP31 was stably knocked down by shRNA against BAP31. (**B**) Results are the means±S.E.M. of three independent experiments, each performed in duplicate. ****p*<0.001, ns, no significance; by Student's t-test.

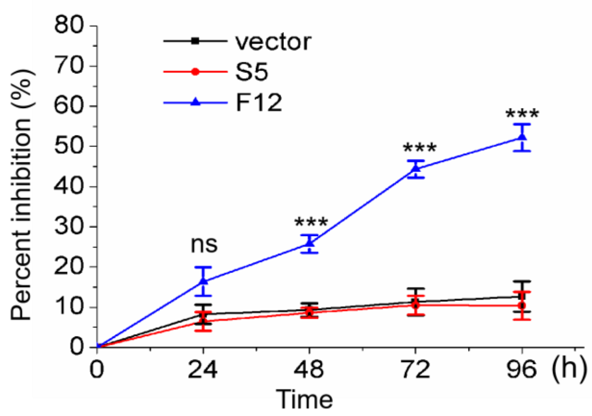

Figure S6. VH-F12 intrabody inhibits cell proliferation of MKN-45 cells. MKN-45 cells were transduced with lentivirus delivered VH-F12 (Lenti-VH-F12). Controls consisted of a Lenti-VH-irrelevant (S5) and mock-transduced cells with Lenti-control. 0, 24, 48, 72 and 96 h after transduction, inhibition of cell proliferation was evaluated by MTT assay. Results are the means±S.E.M. of three independent experiments, each performed in duplicate. ns, no significance, ***P<0.001; by Student's t-test.

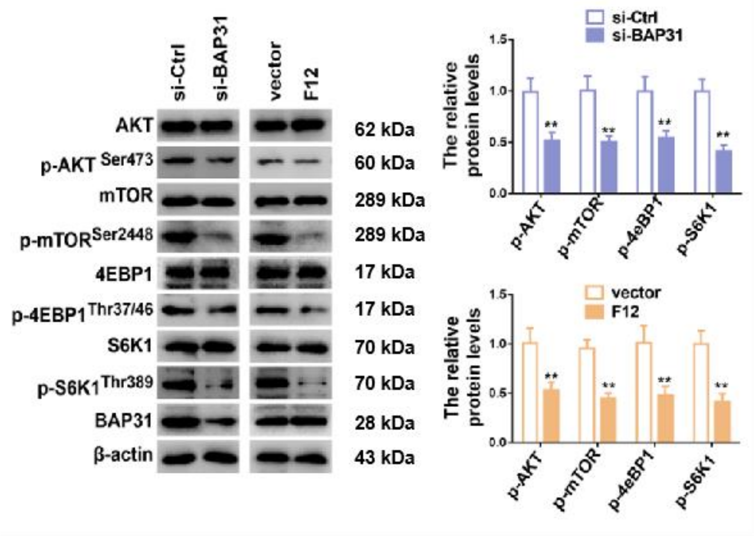

Figure S7. Effect of VH-F12 on PI3K/Akt/mTOR signaling pathway. Western blotting showing expression levels of proteins related to PI3K/Akt/mTOR signaling pathway following BAP31 knockdown by siRNA or VH-F12 expression in MKN-45 cells. Eight signal transduction molecules (Akt, p-Akt, mTOR, p-mTOR, 4EBP1, p-4EBP1, S6K1 and p-S6K1) involved in PI3K/Akt/mTOR signaling pathway were assessed. Bar graphs were obtained by normalizing to actin. The results are the means ± S.E.M. of three independent experiments, each performed in duplicate. **p<0.01; by Student’s t-test.

Table S1: List of antibodies used in this study

Antibody	Isotype	Cat#	Source	Dilution	RRID
BAP31	Rabbit IgG	ab37120	Abcam	1:1000	AB_725626
BAP31	Mouse IgG	SAB1406931	Sigma	1:1000	AB_10740907
β-actin	Mouse IgG	A1978	Sigma	1:10000	AB_476692
Anti-HA	Mouse IgG	H9658	Sigma	1:10000	AB_260092
Calnexin	Rabbit IgG	C4731	Sigma	1:1000	AB_476845
Beclin I	Rabbit IgG	ab62557	Abcam	1:1000	AB_955699
ATG5	Mouse IgG	A2859	Sigma	1:1000	AB_1840708
LC3α, β	Rabbit IgG	ab128025	Abcam	1:1000	AB_11143008
EpCAM	Rabbit IgG	ab8666	Abcam	1:1000	AB_306701
EpCAM	Rabbit IgG	ab223582	Abcam	1:1000	AB_2762366
Integrin β1	Mouse IgG	ab24693	Abcam	1:1000	AB_448230
AKT	Rabbit IgG	SAB4500797	Sigma	1:1000	AB_10745482
		ab64148	Abcam	1:1000	AB_1141028
p-AKT^{Ser473}	Rabbit IgG	ab81283	Abcam	1:1000	AB_2224551
mTOR	Rabbit IgG	ab2732	Abcam	1:1000	AB_303257
p-mTOR^{Ser2448}	Rabbit IgG	ab131538	Abcam	1:1000	AB_11154877
4EBP1	Rabbit IgG	ab2606	Abcam	1:1000	AB_2097994
p-4EBP1^{Thr46}	Rabbit IgG	ab27792	Abcam	1:1000	AB_2097992
S6K1	Rabbit IgG	ab32529	Abcam	1:2000	AB_777800
p-S6K1^{Thr389}	Rabbit IgG	ab2571	Abcam	1:1000	AB_303169
Anti-Mouse IgG-Peroxidase	Goat IgG	A9044	Sigma	1:50000	AB_258431
Anti-Rabbit IgG-Peroxidase	Goat IgG	A9169	Sigma	1:50000	AB_258434

CST: cell signaling technology.
